# Electrospun Fibrous Membranes with Super-large-strain Electric Superhydrophobicity

**DOI:** 10.1038/srep15863

**Published:** 2015-10-29

**Authors:** Hua Zhou, Hongxia Wang, Haitao Niu, Tong Lin

**Affiliations:** 1 Institute for Frontier Materials, Deakin University, Geelong, VIC 3216, Australia

## Abstract

Large-strain elastic superhydrophobicity is highly desirable for its enhanced use performance and functional reliability in mechanically dynamic environments, but remains challenging to develop. Here we have, for the first time, proven that an elastic fibrous membrane after surface hydrophobization can maintain superhydrophobicity during one-directional (uniaxial) stretching to a strain as high as 1500% and two-direction (biaxial) stretching to a strain up to 700%. The fibrous membrane can withstand at least 1,000 cycles of repeated stretching without losing the superhydrophobicity. Stretching slightly increases the membrane air permeability and reduces water breakthrough pressure. It is highly stable in acid and base environments. Such a permeable, highly-elastic superhydrophobic membrane may open up novel applications in membrane separation, healthcare, functional textile and energy fields.

Superhydrophobic surfaces with large contact angle and low contact angle hysteresis have shown enormous potential for applications in areas as diverse as self-cleaning, anti-sticking, anti-fogging, anti-icing, anti-pollution, anti-fouling, drag reduction, buoyancy enhancement, corrosion-resistant, and liquid separation^1^,^2^,^3^,^4^,^5^,^6^,^7^,^8^,^9^,^10^,^11^,^12^. For practical applications, superhydrophobic surfaces are often required to be robust enough against harsh conditions. Depending on the application, a superhydrophobic surface is desirable to withstand physical abrasion, repeated washing, organic solvents, acid/base, chemical agents, biological substances, strong radiation or heat. Besides, the resilience to mechanical deformation (e.g. stretching or compression) is also potentially important for superhydrophobic materials to work on dynamic deformation. It can effectively avoid the loss of superhydrophobicity during or post deformation, which is the main source to cause the faults of protection, self-cleaning, or anti-fouling functions, or incidents in oil-water separation. In this regard, rendering superhydrophobic materials with ability to maintain superhydrophobicity during frequent deformation and shape recovery, i.e. elastic superhydrophobicity, would greatly enhance the functional reliability, durability and application performance. Furthermore, large-strain elastic superhydrophobicity enables to withstand extreme deformation such as those can lead to overstretching during movement or exercise or deforming under high-speed impacts, opening up opportunities for development of novel applications especially in healthcare, biomedical, functional textile, and energy areas.

Deformable superhydrophobicity have been prepared for example on a commercial polyurethane sponge^13^, polyurethane fiber mat^14^, polydimethylsiloxane (PDMS) film^15^, three-dimensional silica nanostructure^16^, and elastic aerogel^17^ (see a detail summary in supporting information, Table S1). However, most of the deformable superhydrophobic materials have a working strain below 100% (only one have a strain of 300%), they were prepared by a complex multiple-step procedure, and only single-direction elasticity was reported. For practical applications, materials often involve multi-directional deformation. However, there is no data for elastic superhydrophobic materials under multidirectional deformation reported in research literature.

Herein, we have, for the first time, proven a fibrous membrane with incredibly high elastic superhydrophobicity on a large strain (1500% for uniaxial stretching and 700% for biaxial stretching). This novel fibrous membrane was prepared by electrospinning of poly(styrene-butadiene-styrene) (SBS) and by subsequent hydrophobization with a fluoroalkyl silane (FAS). It maintains its superhydrophobicity even after 1,000 cycles of stretching to uniaxial strain as high as 1500% or biaxial strain of 700%. To our knowledge, this should be the highest elasticity that has even been reported for superhydrophobic materials. The fibrous membrane is air-permeable with a large water breakthrough pressure, as well it is very stable in strong acid or alkaline environment.

## Results

**Figure 1:**
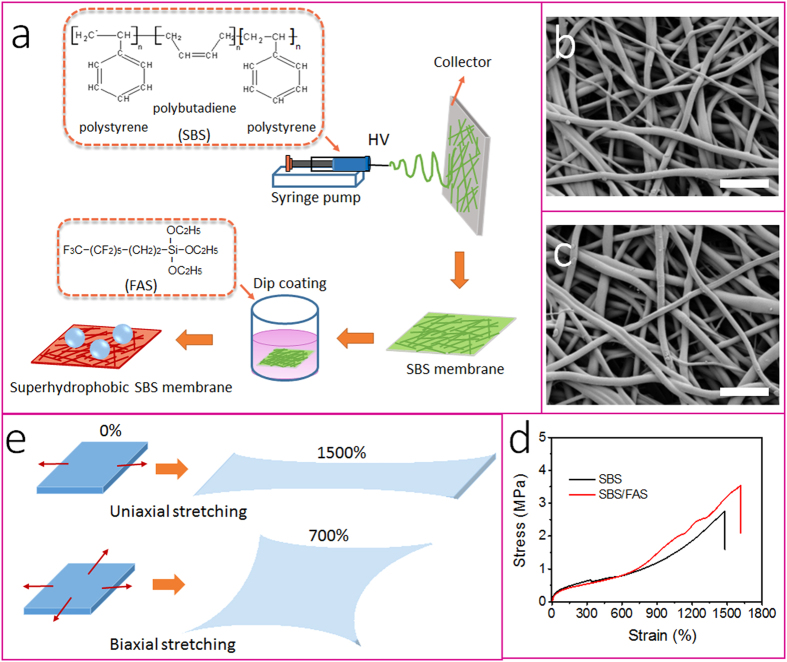
(**a**) Procedure for preparing superhydrophobic fibrous membrane, (**b,c**) SEM images of (**b**) electrospun SBS fibers and (**c**) FAS-modified SBS fibers (scale bar: 10 μm); (**d**) strain-stress curves of the fibrous membranes, (**e**) schematic illustration to show the fibrous membrane under uniaxial and biaxial stretching.

Figure 1a depicts the procedure to prepare FAS modified SBS fibrous membrane. An electrospinning technique was employed to process a SBS-DMF/THF solution into a fibrous membrane. After electrospinning, the fibrous membrane (thickness around 300 μm) was modified with FAS through a dip-coating method.

Figure 1b shows the SEM image of the electrospun fibrous membrane. All fibers look uniform without bead. Similar to common electrospun fibers, the as-spun SBS fibers had a randomly-orientated fibrous morphology in the membrane. The average diameter of the SBS fibers was 2.5 μm. After FAS modification, the fibers showed almost no change in morphology (Fig. 1c). We also performed FTIR-ATR and XPS measurements to confirm that fluorinated alkyl chains attached the fiber surface after FAS treatment (see Supporting Information).

The SBS membrane was found to have high elasticity. It can be stretched many times longer than its original length, and the stretched membrane can retract to its original shape rapidly once the stretching force is released. Figure 1d shows the stress-strain curves of the SBS fibrous membranes. Both the as-spun SBS and the FAS modified SBS fibrous membranes show very large break strain (*ε*_max _= Δ*L*_max_/*L*·100%), being 1480% and 1610%, respectively, although the tensile strength was just several MPa. The FAS-modified SBS fibrous membrane showed higher tensile strength and break strain in comparison to the as-spun SBS fiber membrane, indicating the increase in toughness. Apart from the uniaxial stretching, biaxial stretching was also performed on the membrane (see the stretching illustration in Fig. 1e). The membrane can be stretched in biaxial mode to a strain in both directions (i.e. biaxial strain) as large as 700%. To our knowledge, 1610% and 700% should be the highest uniaxial and biaxial strains that have ever been reported for electrospun fibrous membranes so far.

**Figure 2:**
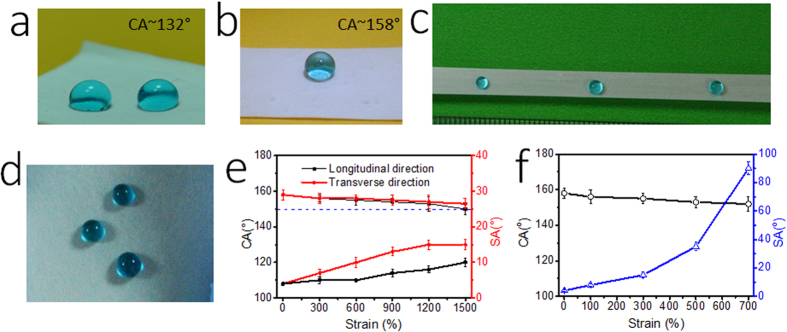
(**a–d**) Blue-dyed water drops on: (**a**) SBS fiber membrane, (**b**) FAS-modified SBS fiber membrane, (**c**) FAS-treated fiber membrane under 1500% uniaxial strain, (**d**) FAS-treated fiber membrane under 700% biaxial strain; (**e**) CA and SA change in longitudinal and transverse directions under different uniaxial stretching levels, (**f**) CA and SA changes with biaxial strain.

Figure 2a,b show the wetting behavior of the SBS fibrous membranes. The as-spun SBS was hydrophobic with a water contact angle (WCA) of 132°. After FAS modification, the membrane turned superhydrophobic with a WCA of 158° and a sliding angle (SA) as low as 5°. More interestingly, the FAS-modified SBS membrane maintained the superhydrophobicity under stretching. As seen in Fig. 2c,d, water drops keep the sphere shape on both the uniaxially-stretched (strain 1500%) and the biaxially stretched (biaxial strain 700%) fibrous membranes. The membranes after stretching and retracting to its original shape still retained the superhydrophobicity (see more results in Supporting Information).

Figure 2e,f show the CA and SA changes with strain level. It was interesting to note that the FAS-modified SBS membrane under uniaxial stretching showed an anisotropic wetting behavior (Fig. 2e). When the strain was larger than 300%, the CA measured in the longitudinal direction became lower than that in the transverse direction, and the CA difference increased with increasing the strain level. At the uniaxial strain of 1500%, the average CAs in the transverse and the longitudinal directions were 153° and 150°, respectively. Larger difference was noted on SA. On the uniaxially stretched fibrous membrane, SA along the longitudinal direction was lower than that along the transverse direction. At the strain of 1500%, the fibrous membrane had a SA of 10° and 15° along the longitudinal and the transverse directions.

In contrast, water on biaxially-stretched fibrous membrane showed an isotropic wetting feature. The biaxial stretching had larger effect on membrane surface wettability, although the membrane under biaxial stretching was still above 150° in CA (e.g. 152° at the biaxial strain 700%), whereas the SA increased with the biaxial strain on a larger extent (Fig. 2f). At the biaxial strain of 300%, the SA was 16°, and the SA increased to 38° at 500% strain. In such a large SA, the water drop stuck firmly on the membrane surface.

**Figure 3:**
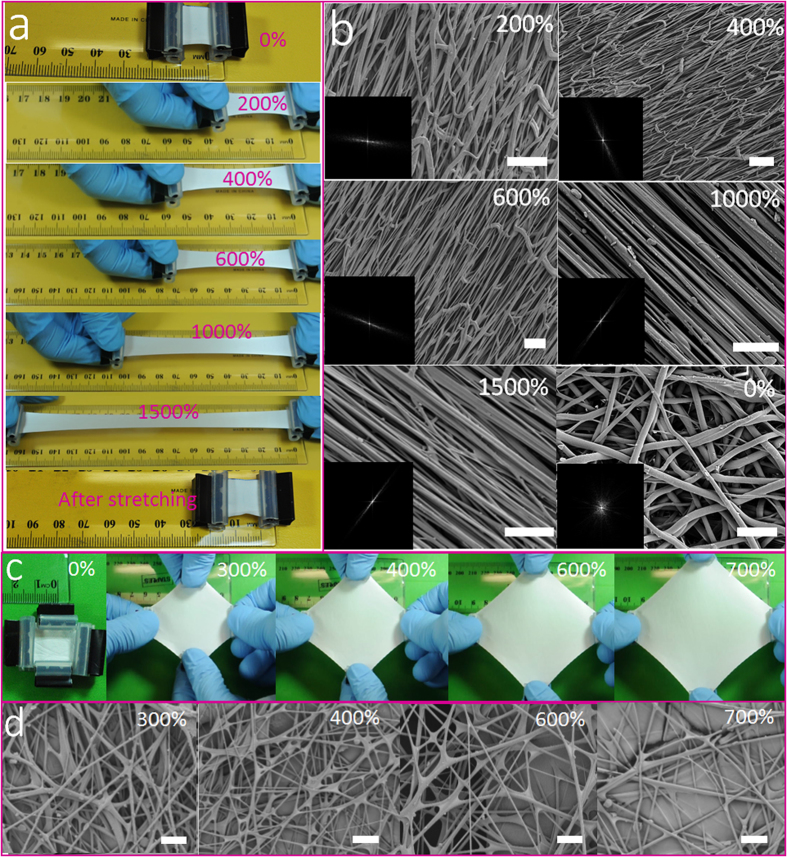
(**a**) Still frames taken from a video to show uniaxially stretching a FAS-treated fibrous membrane (ε = Δ*L/L* × 100%) (*L* is the length of the membrane), (**b**) SEM images of FAS-treated fibrous membrane under a uniaxial strain from 0% to 1500% (scale bar: 10 μm; the insert images are the FFT frequency images), (**c**) still frames showing biaxial stretching SBS/FAS membrane to two-directions from 0% to 700%, (**d**) SEM images of biaxial stretched membrane under different strain levels (scale bar: 10 μm).

Figure 3a,b show photos and SEM images of the FAS-modified SBS fibrous membrane under uniaxial stretching. With increasing the strain level, the fibers tended to align along the drawing direction. The insert images in Fig. 3b show fiber alignment characterized based on the SEM images. With increasing the strain level, the white pixel distribution changed into an elliptic pattern, attributable to the high alignment along the stretching direction. When the strain was reduced to 0% strain, the white pixel turned into a symmetric, circular pattern around the origin in the FFT image, which is similar to the non-stretched state (see Supporting Information).

The morphology of fibrous membrane under biaxial stretching was also examined. As seen in Fig. 3c,d, biaxial stretching leads to larger pores due to the two-dimensional expansion of the fibrous membrane. Different to the uniaxial stretching, the biaxial stretching maintained the randomly-orientated fibrous feature.

Apart from the morphology change, the fiber diameter and membrane thickness also changed with stretching (see Supporting Information). Both fiber diameter and membrane thickness decreased with increasing the strain level. Uniaxially stretching the FAS-modified SBS membrane from 0% to 1500% strain decreased the average fiber diameter from 2.45 μm to 0.45 μm, and the membrane thickness decreased accordingly from 300 μm to 145 μm. For the biaxially stretched fibrous membrane, the average fiber diameter and membrane thickness decreased by 66% (from 2.45 μm to 0.83 μm) and 62% (from 300 μm to 115 μm), respectively, when the biaxial strain increased from 0% to 700%.

**Figure 4:**
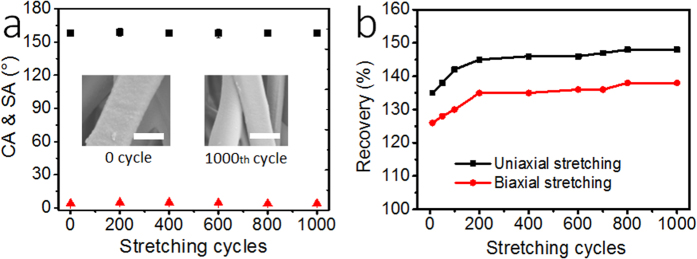
Effect of stretching cycles (strain 0% → 1500% → 0% for each cycle) on WCA and SA of the uniaxially-stretched FAS-SBS fiber membrane (the inserts are SEM images of the non-stretched fibers and the ones after 1,000 cycles of stretching, scale bar: 2 μm), (b) length recovery after uniaxial or biaxial stretching.

The fibrous membranes showed an excellent elasticity. It can be stretched repeatedly to large strains for many times. Figure 4a shows the effect of repeated uniaxial stretching on the superhydrophobicity of the FAS-modified SBS fibrous membrane. During test, the membrane was stretched from strain zero to 1500% and then the stretching force was released to allow the membrane retract to the freestanding state. After 1,000 cycles of stretching and retracting, the average CA and SA of the fibrous membrane were kept almost unchanged, at 156° and 4°, respectively. The inset images in Fig. 4a show the morphology of the fibers before and after 1,000 cycles of stretching. The fibers after so many cycles of stretching still kept their morphology. Similarly, the effect of repeated biaxial stretching on membrane wettability was also examined. In each cycle, the membrane was stretched biaxially from strain 0% to 700%, and then retracted to freestanding state. After 1,000 cycles of biaxial stretching and retracting, the CA and SA were almost unchanged as well.

Restoration ratio (L_after_/L_before_) was employed to characterize the effect of stretching cycles on membrane dimension. As shown in Fig. 4b, the fibrous membrane after the first cycle of uniaxial stretching is indeed elongated, with the length restoration ratio increased to 135%. However, further stretching only led to very little change in membrane length. After 200 cycles of stretching, the membrane length did not change with further stretching. For the biaxial stretching, the membrane increased to 125% of the original length after the first stretching cycle, and the overall length in both directions increased only by 10% in the further 200 cycles of stretching. When the stretching cycle increased from 200 to 1,000, the recovery ratio showed no obvious change (Fig. 4b). The unrecovered elongation at the initial stretching cycle was attributed to the rearrangement of the fibers within the membrane after stretching. This phenomenon is similar to PDMS fiber membranes previously reported by our group^18^.

To examine the effect of FAS on SBS surface hydrophobicity, we prepared a dense flat SBS film by casting the SBS solution on a glass substrate and then treated the film surface with FAS-ethanol (i.e. the same procedure to treat the SBS fibrous membrane). As expected, the cast SBS film after FAS modification increased the WCA from 81.6° to 111.3°. FAS coating had a thickness around 60 nm (measured by AFM imaging see Supporting Information).

To verify the elasticity, we tested the stretching performance of the FAS-modified SBS cast film and its effect on surface wettability. As expected, the FAS modified SBS cast film showed repeatable elasticity with the break strain greater than 1000% (see strain-stress curves in Supporting Information). At 1000% strain, the WCA value was 112°.

**Figure 5:**
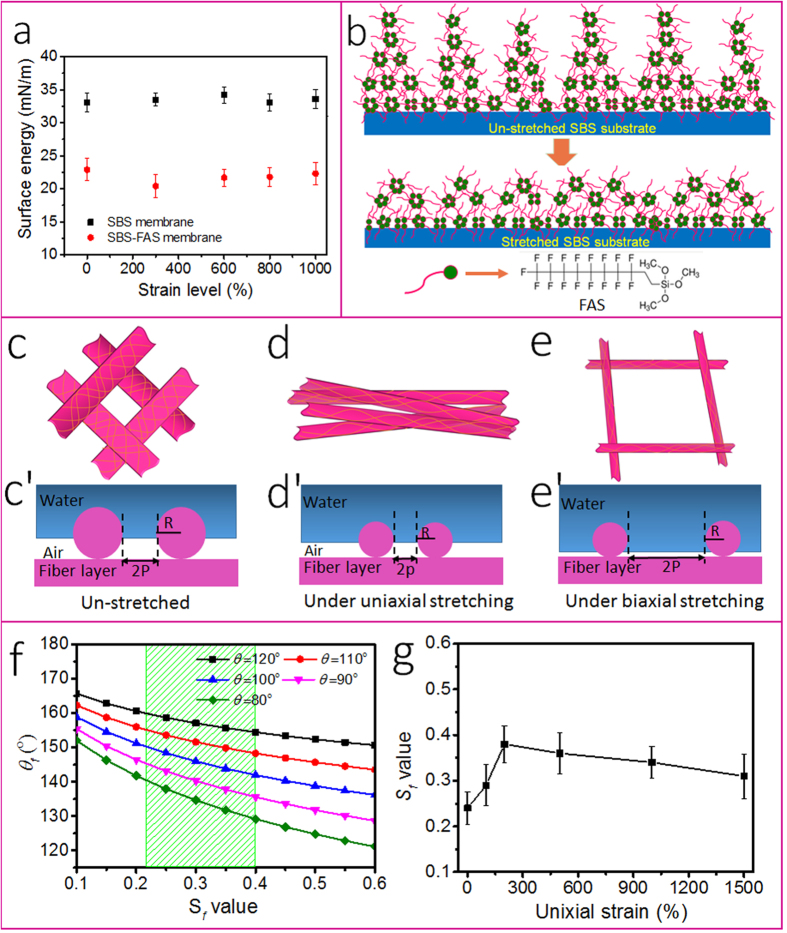
(**a**) Surface energy of the SBS cast films at different strain values, (**b**) schematic illustration of the FAS molecules on SBS substrate, **(c–e**) schematic illustration of FAS-treated fibrous membrane: (**c**) without stretching, (**d**) under uniaxial stretching, and (**e**) under biaxial stretching; (**c–e**) illustration of wetting fibrous membranes at different states; (**f**) estimated *θ*_*f*_ ~ *S*_*f*_ relationship, (**g**) experimental result on the relationship between uniaxial strain and *S*_*f*_.

To find out the source of the stable hydrophobicity under stretching, we also tested the effect of stretching on the surface free energy of the film using the Wu’s model^19^. As seen in Fig. 5a, the FAS modified SBS film has a surface energy around 22 mN/m (33 mN/m for the uncoated SBS membrane); the surface energy was almost unchanged with increasing the strain level (see details in Supporting Information). The FTIR spectra indicated that stretching had almost no effect on the surface chemistry of the FAS modified SBS film (Supporting information). At 1000% strain, the stretched film showed almost identical FTIR to the un-stretched one.

In addition, the FAS-modified SBS fibrous membrane was permeable to air and moisture, and it had a large breakthrough pressure to liquid water (see supporting information). Stretching led to increase in air permeability but decrease in breakthrough pressure. The first uniaxial stretching from 0% to 1500% increased the air permeability from 1.23 cm^3^/cm^2^/s to 7.80 cm^3^/cm^2^/s, and meanwhile decreased the breakthrough pressure from 4.98 mH_2_O to 1.55 mH_2_O. Further stretching cycles had very small effect on the air permeability and breakthrough pressure (see detail results in Supporting Information). In addition, the FAS-modified SBS fibrous membrane was found to be very stable in acid and base environments. After immersing in aqueous H_2_SO_4_ (pH = 1) or KOH (pH = 14) solution at room temperature for 2 days, then rinsing the membrane with water, and finally drying it at room temperature, the fibrous membrane still maintained its original superhydrophobicity (see Supporting Information).

## Discussion

SBS is a non-polar block copolymer comprising three segments (i.e., two polystyrene and one polybutadiene) in the backbone. It is hydrophobic and has high elasticity. FAS is a polar molecule comprising a hydrolysable silane head and a fluoroalkyl chain. It is often used for hydrophobic treatment. Based on the chemical structures, the interaction between FAS and SBS should be mainly on physical basis. Figure 5b illustrates the possible interactions between SBS and FAS molecules. Single molecule FAS cannot stay stably either on SBS surface or in SBS matrix due to the mismatched polarity between the two materials. Alternatively, the silane heads could hydrolyze and aggregate together to form a core and the fluoroalkyl chains expose to the surface. In this way, FAS molecules could form micelle-like structures or bimolecular layers on SBS surface. The high non-polar affinity between the fluoroalkyl chains and SBS could enable the fluoroalkyl chains partially migrate into the SBS substrate, enhancing the adhesion. As a result, FAS forms a stable coating on SBS surface. Since the 60 nm thickness is fairly larger than the size of FAS molecule (less than 1 nm), the FAS aggregates should be in multilayer on SBS.

Based on our experiment results on the FAS-modified dense SBS film, the stable hydrophobicity under stretching can be explained by that: 1) FAS forms multilayer low-surface-energy FAS aggregates on the SBS substrate, 2) the layer next to the SBS shows strong binding with SBS through the fluoroalkyl chains, 3) under stretching the upper layer FAS aggregates could slip to cover the exposed SBS surface, lowering the surface energy.

It is known that superhydrophobicity comes from rough surface with low surface free energy. The SBS fibrous membrane has an intrinsically rough surface originated from the randomly-orientated fibrous texture and FAS treatment lowers the surface energy, making the FAS-modified fibrous membrane have superhydrophobicity.

Figure 5c,e schematically illustrate the proposed stretching states. Under uniaxial stretching, the fibers turned aligning with a decrease in the inter-fiber distance (Fig. 5d), while the inter-fiber distance increased significantly under biaxial stretching (Fig. 5e). Figure 5c’ illustrate the corresponding cross-sectional geometries of the fibers on the top of the membrane and their wetting states. Water on the non-stretched fibrous membrane is typically in a Cassie-Baxter wetting state because of the air bubbles trapped within the fibrous membrane and the low surface energy of the fibers. The apparent WCA can be estimated by the equation^20^:


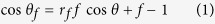


where *θ* is the WCA of the fiber material, *r*_*f*_ is the roughness of surface that contacts with liquid, and *f* is the *f*ractional area of liquid contacting with the solid surface. The relationship between *θ*_*fabric*_ and *θ* can be estimated as^21^:


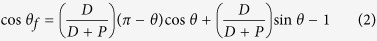


Here, *D* is the fiber diameter (*D* = 2R) and *P* is the inter-fiber distance. For the fibrous membrane, the surface fractional area can be defined as dimensionless spacing factor *S*_*f*_ *=* *D/P*^22^. Therefore, the equation (2) can be modified as:


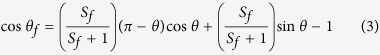


Figure 5f shows *θ*_*f*_ ~ *S*_*f*_ relationship at a few different *θ* values.

Based on the SEM images of the fibrous membrane under various stretched states, we measured their *S*_*f*_. As shown in Fig. 5g, the measured *S*_*f*_ changes with the variation of the uniaxial strain level. When the strain was less than 200%, *P* reduced owing to the fiber alignment whereas the *D* value did not change much. This led to increase of the *S*_*f*_ value to 0.38 ± 0.04 at strain level 200%. With further increasing the strain level, the *P* maintained almost unchanged, while the *D* value decreased. This leads to decrease in *S*_*f*_ value to 0.31 ± 0.05 at strain 1500%.

We also estimated the WCA of the fibrous membranes (i.e. *θ*_*fabric*_) at different stretching states using the equation (3) and the *θ* and *S*_*f*_ same to the experimental values (based on the cast films for *θ*). At the un-stretched state (*S*_*f*_ = 0.24, *θ* values 81.6° and 111.3° for the cast and the FAS-modified SBS films, respectively), the calculated *θ*_*fabric*_ values were 138.2° and 153.8°, respectively, for the as-spun SBS and FAS-modified SBS fibrous membranes (Fig. 5f). The calculation was very close to experimental results, being 132° and 158° (Fig. 2e). For the FAS-modified SBS fibrous membrane at 1500% strain (*S*_*f*_ = 0.31), the calculated *θ*_*fabric*_ was 152°, which is also close to our tested CA value (150° and 153° in longitudinal and transverse directions, respectively) (Fig. 2e).

Under biaxial stretching, the pore sizes of the fibrous membrane increased greatly due to the two-dimensional structural expansion. This led to increased *P* but decreased *D*, hence decreased *S*_*f*_ value. At the biaxial strain of 700%, the *S*_*f*_ measured based on SEM image was 0.14 ± 0.05. At such a *S*_*f*_ and almost unchanged *θ*, the fibrous membrane estimated was 158° (experiment value, 152°) (Fig. 2f). Therefore, the highly elastic superhydrophobicity of the FAS-modified SBS fibrous membrane should come from the small change in *S*_*f*_ and *θ* under stretching.

The SA of a superhydrophobic surface is mainly determined by surface morphology^23^,^24^. Under uniaxial stretching, the fibers turned to orientate along the stretching direction, companied by a decrease in the inter-fiber distance and the fiber diameter. The fiber alignment makes the surface morphology resemble a grooved surface. A main effect of the surface grooves on the superhydrophobicity is anisotropic wettability^25^.

Under biaxial stretching, the fibrous membrane shows increased inter-fiber distance (Fig. 5e,e’), which resulted in a decreased surface roughness. Under large strain level, the water droplet even pinned in the membrane’s matrix, which may lead the water droplet stick on the surface.

## Conclusion

We have proven that a fibrous membrane prepared by electrospinning of SBS and by subsequent modification with a FAS shows highly elastic superhydrophobicity. The fibrous membrane maintains the superhydrophobicity at a large strain, even after a thousand cycles of stretching. Stretching slightly increases the membrane air permeability but reduces the water breakthrough pressure. The membrane is also highly stable in acid and base environments. Our elastic superhydrophobic fibrous membrane may find applications in membrane separation, healthcare, functional textile and energy areas.

## Methods

### Materials

Poly(styrene-butadiene-styrene) (SBS, KRATON® D 1102 B Polymer with a polystyrene content of 29.5%) was purchased from Kraton company (United States), 1H,1H,2H,2H-perfluorodecyltriethoxysilane (C_16_H_19_F_17_O_3_Si) (FAS) was provided by Plastral Pty Ltd. Phenolphthalein, tetrahydrofuran (THF), dimethylformamide (DMF), ethanol and ammonia (28% in water) were purchased from Aldrich. All chemicals were used as received. SBS solution was prepared by dissolving SBS in a solvent mixture of THF and DMF (70/30, v/v). The SBS concentration in the solution was controlled at 17.0 wt%.

### Electrospinning

A purpose-made electrospinning setup was employed for making SBS nanofibrous membrane^26^. During electrospinning, the SBS solution was charged with high voltage using a power supply (ES30P, Gamma High Voltage Research), and a metal plate was used as collector. The flow rate of the polymer solution was controlled by a digital syringe pump (KDS-200, KD Scientific). The applied voltage, flow rate of the SBS solution, and spinning distance were controlled at 20.0 kV, 3 ml/h and 17 cm, respectively.

### Surface modification

The as-spun SBS membrane was immersed in an ethanol solution containing 2% (v/v) FAS. After 2 minutes, the membrane was taken out and then dried at room temperature for 1 hour.

### Preparation of SBS films

SBS film was prepared by using the same SBS spinning solution through a casting method, briefly, 5 ml SBS solution was poured onto a glass slide and air dried overnight. The FAS modified cast SBS membrane was prepared through the same coating procedure aforementioned above. The SBS films for AFM imaging was prepared by spin-coating 0.5 ml SBS solution (1.0% w/v) (DMF:THF = 7:3) onto a silicon wafer (800 rpm for the first 5 s and thereafter 2500 rpm for 20 s). The spin-coated SBS film was then partially modified with FAS through a dip-coating method similar to the procedure for treatment of SBS fibrous membranes and cast films.

### Acid/base stability

The test was performed at 20 ± 2 °C. H_2_SO_4_ (pH = 1) and KOH (pH = 14) solutions were used to test the chemical stability. Briefly, immersing the samples in the acid and base solutions, respectively. After 2 days, the samples were taken out and then dried at room temperature.

### Characterizations

Scanning electron microscopy (SEM) images were taken using SEM Supra 55 VP. The fiber diameter, inter-fiber spacing were measured by Imagepro + 4.5 and over 50 read were taken for each test. The fiber alignments were analyzed by Fourier transform (FFT), the FFT analysis was based on SEM images using ImagePro + 4.5 as well. Contact angle was measured on a contact angle goniometer (KSV CAM 101) using liquid droplets of 5 μL in volume. Air permeability was measured on the FX 3300 air permeability tester according to the standard (SIST EN ISO 9237–1999). Each CA and air permeability were the mean values of 5 measurements. Fourier transform infrared (FTIR) spectra were recorded on a Bruker VERTEX 70 instrument in ATR mode at a resolution of 4 cm^−1^ accumulating 32 scans. X-ray photoelectron spectra (XPS) were collected on a VG ESCALAB 220-iXL XPS spectrometer with a monochromated AL Kα source (1486.6 eV) using samples of ca. 3 mm^2^ in size. The X-ray beam incidence angle is 0° with respect to the surface normal, which corresponds to a sampling depth of ca. 10 nm. The obtained XPS spectra were analyzed by the CasaXPS software. Tensile property was measured on an Instron Tensile Tester at controlled environmental temperature of 20 ± 2 °C and relative humidity of 65 ± 2%. Surface morphology and the cross section of the membranes were measured using atomic force microscope (AFM, Asylum Research). The thickness of the membrane was measured using a thicknesses tester (Digimatic Indicator, Mitutoyo). Breakthrough pressure was measured using customer-built equipment comprising a fluid-feeding system with a flow rate controller, a pressure gauge, and a sample holder. During measurement, the fluid was loaded on one side of the fibrous membrane at a flow rate of 20 ml/min and the minimum pressure at which the fluid started passing through the membrane was recorded as the breakthrough pressure (see the diagram in the supporting information).

## Additional Information

**How to cite this article**: Zhou, H. *et al.* Electrospun Fibrous Membranes with Super-large-strain Electric Superhydrophobicity. *Sci. Rep.* **5**, 15863; doi: 10.1038/srep15863 (2015).

## Supplementary Material

Supporting Information
