# Supplementary material for: Electrospun Fibrous Membranes with Super-large-strain Electric Superhydrophobicity
Source: Sci Rep. 2015 Oct 29;5:15863. doi: 10.1038/srep15863 (PMC4625365; doi:10.1038/srep15863)
Supplement: Supporting Information [file srep15863-s1.doc]

Supporting Information

Electrospun Fibrous Membranes with Super-large-strain Electric Superhydrophobicity

Hua Zhou, Hongxia Wang, Haitao Niu, Tong Lin*

Institute for Frontier Materials, Deakin University, Geelong, VIC 3216, Australia

*Corresponding author’s Email: [tong.lin@deakin.edu.au](mailto:tong.lin@deakin.edu.au)

Table S1, A summary of deformable superhydrophobic materials

| Substrate |  | Approach |  | Max working strain | ref |
| --- | --- | --- | --- | --- | --- |
| Commercial polyurethane sponge |  | Layer-by-layer deposition of polydopamine and Ag nanoparticles |  | compressive 70% |  |
| Polyurethane fiber mat |  | A three-step process: 1) electrospinning of polyurethane, 2) polymerization to form polyaniline hairy nanostructure, 3) dip-coating with polytetrafluoroethylene solution |  | stretching 300% |  |
| Polydimethylsiloxane (PDMS) film |  | Applying graphene onto a stretched PDMS film substrate |  | 100% |  |
| Three-dimensional silica nanostructure |  | Replicated from a hydrogel template |  | stretching at 100% strain |  |
| Aerogel with hierarchical cellular structure |  | Combining electrospinning with a freeze-shaping technique |  | 80% compression strain |  |
| SBS fiber mat |  | Electrospinning and dip coating with FAS |  | 1500% | Our work |


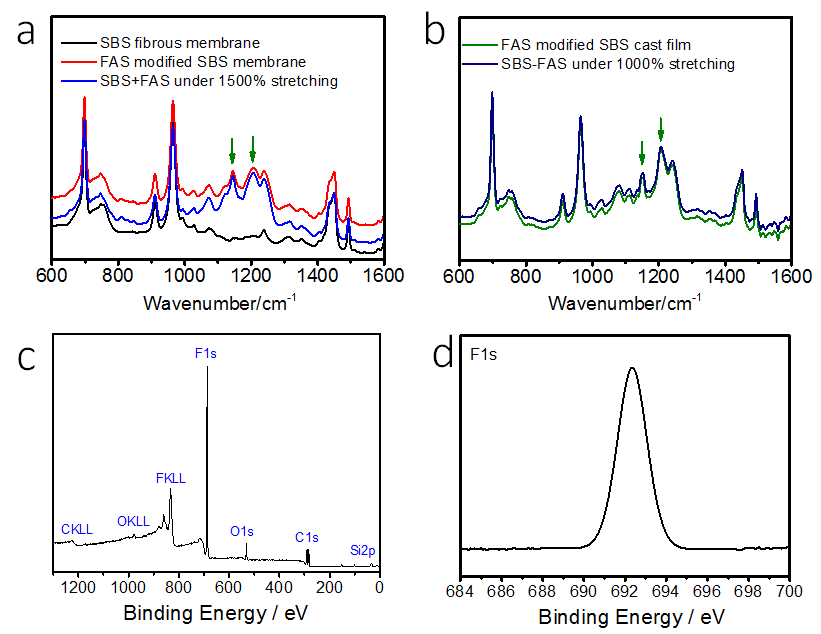


Figure S1. a) ATR-FTIR spectra of the SBS electrospun fibrous membrane before and after FAS modification, b) ATR-FTIR spectra of the SBS casting film before and after FAS modification c) XPS survey spectrum of the FAS coated SBS fibrous membrane, d) XPS high-resolution F1s spectrum of the FAS coated SBS fibrous membrane.


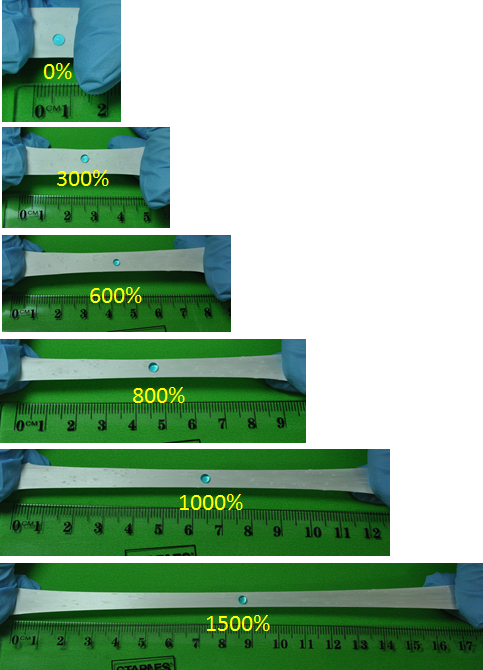


Figure S2. A blue-dyed water drop on the FAS modified SBS fibrous membrane at different strain levels.


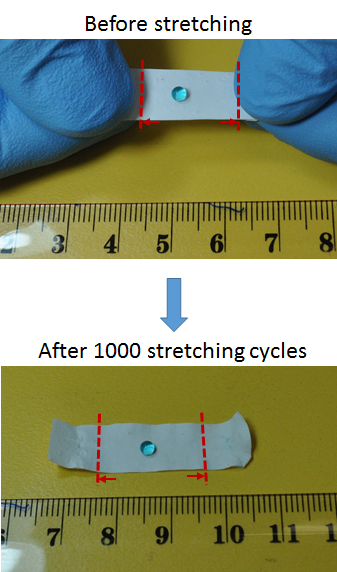


Figure S3. Digital images to show water drop on the fibrous membrane before stretching and after 1,000 cycles of uniaxial stretching (in each cycle, the fibrous membrane was stretched to strain 1500% and then fully relaxed).


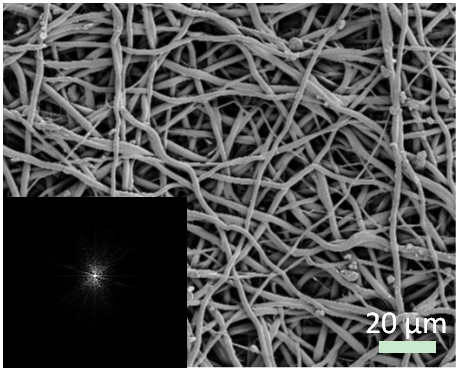


Figure S4. SEM image of the non-stretched SBS fiber membrane (without FAS, the insert images is the FFT frequency image).


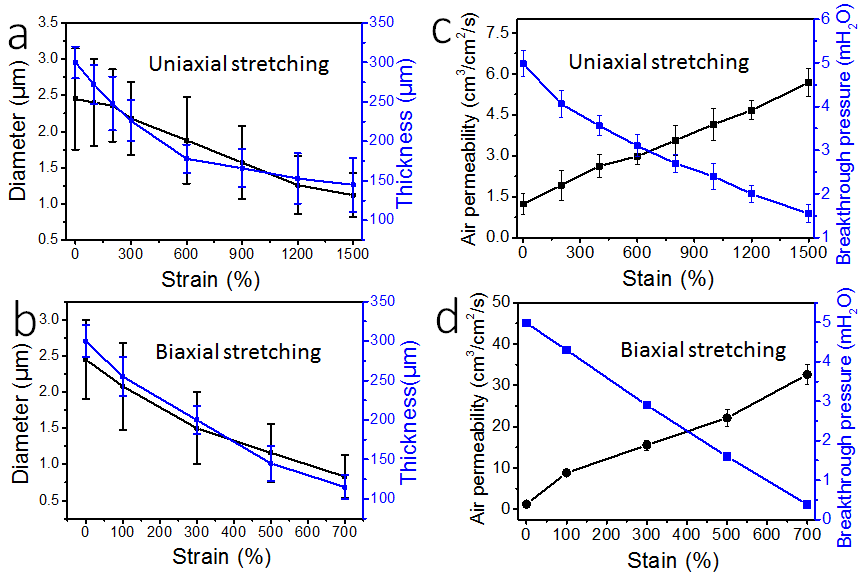


Figure S5. Effects of strain level on, a & b) average fiber diameter and thickness, c & d) air-permeability and breakthrough pressure. (Sample: FAS modified SBS fibrous membrane)


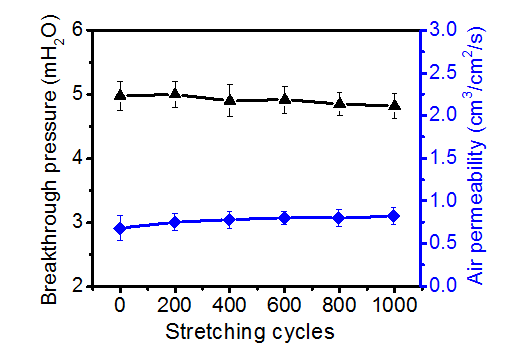


Figure S6. Breakthrough pressure and air permeability change with stretching cycles.


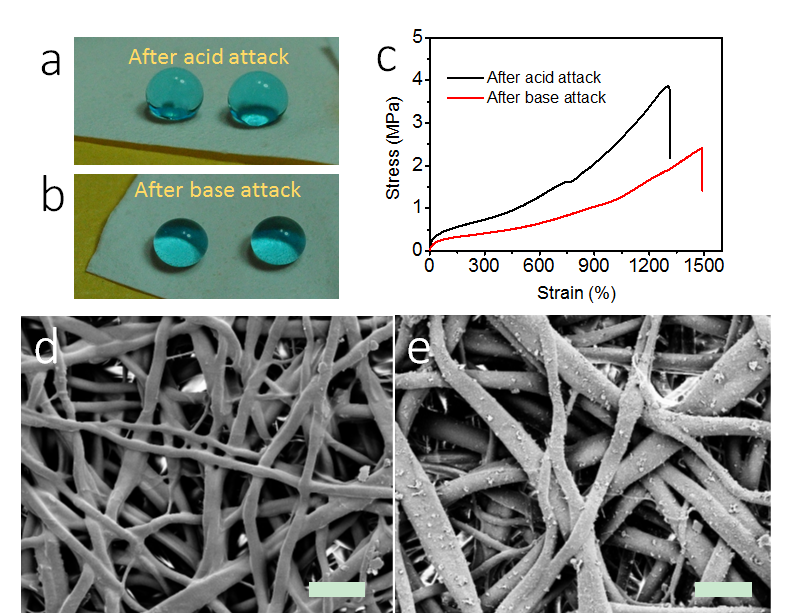


Figure S7. a & b) Blue-dyed water drops on the FAS modified SBS fibrous membrane after immersing in a) acid and b) base solution for 2 days, c) strain-stress curves of the fibrous membrane after acid or base immersion, d & e) SEM images of: d) acid treated membrane, and e) base treated membrane (scale bars: 1 µm).

The membrane after acid or base immersion still had high elasticity. The break strain for the membrane after acid test is 1310%, and 1480% after base test.


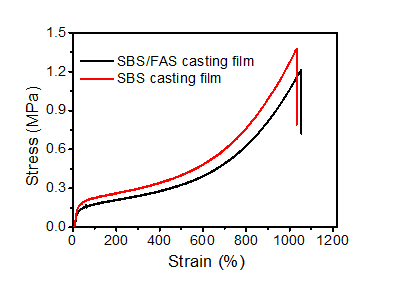


Figure S8. Strain-stress curves of the cast SBS films before and after FAS surface modification.

**Details on calculation of surface energy**

Surface energy was calculated based on the Wu’s method according to equation below:


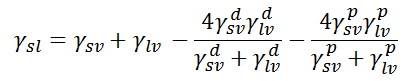
 (1)

According to Young’s equation:


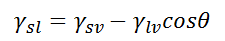
 (2)

The solid/liquid interfacial energy (γ_sl_) can be calculated from solid/gas interfacial energy (γ_sv_), liquid surface tension (γ_lv_), and the contact angle (*θ*) of the liquid on the solid surface. The surface tension of the liquid in dispersive (γ_lv_^d^) and polar (γ_lv_^p^) components are known, while the surface energy for the solid substrate in dispersive (γ_Sv_^d^) and polar (γ_Sv_^p^) components are unknown. By measuring *θ* to tetraethylene glycol and deionized water, γ_Sv_^d^ and γ_Sv_^p^ can be obtained. In this way, the surface energy of coatings was calculated as listed in Table S2.

**Table S2** Surface energy of coating materials

|  | Strain | Avg. *θ*  Water [°]* | Avg. *θ*  ethylene glycol [°]* | γ^polar^  [mN/m] | γ^dispersive^  [mN/m] | γ^total^  [mN/m] |
| --- | --- | --- | --- | --- | --- | --- |
| SBS films | 0% | 84 | 52 | 21.9 | 11.2 | 31.1 |
|  | 300% | 85 | 51 | 23.3 | 10.2 | 33.5 |
|  | 600% | 86 | 50 | 24.9 | 9.2 | 34.1 |
|  | 800% | 84 | 52 | 21.9 | 11.2 | 33.1 |
|  | 1000% | 85 | 52 | 23.3 | 10.2 | 33.5 |
| FAS Modified SBS films | 0% | 111 | 82 | 22.2 | 0.7 | 22.9 |
|  | 300% | 110 | 84 | 15.0 | 5.4 | 20.4 |
|  | 600% | 109 | 82 | 20.2 | 1.5 | 21.7 |
|  | 800% | 109 | 81 | 19.8 | 2.0 | 21.9 |
|  | 1000% | 112 | 78 | 21.1 | 2.0 | 23.1 |

* γ_lv_^d^ =21.8 mN/m and γ_lv84_^p^= 51.0 mN/m for water; γ_lv_^d^ =32.8 mN/m and γ_lv_^p^= 16.0 mN/m [ref: Wu, S., *Calculation 85of interfacial tension in polymer systems.* Journal of Polymer Science Part C: Polymer Symposia, 1971. **34**(1): 19-30]


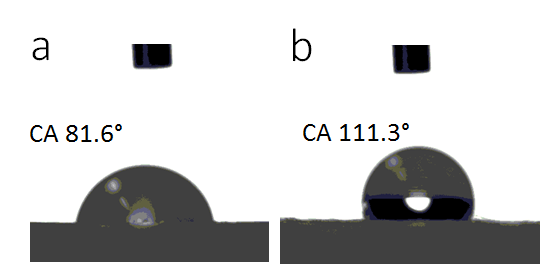


Figure S9. CA values of: a) SBS cast film, and b) FAS modified SBS cast film.


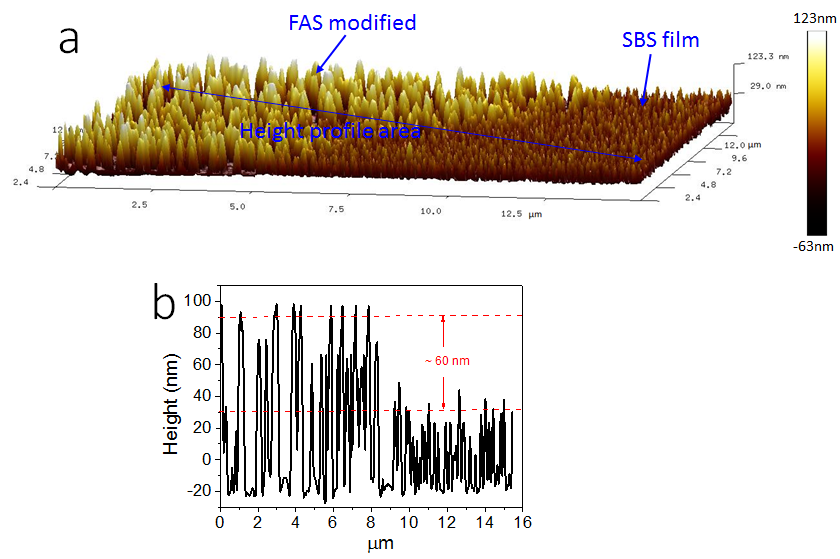


Figure S10. a) AFM image of the spin-coated SBS film partially modified with FAS, and b) the height profile along the line marked in a.

**Details on breakthrough pressure measurement**


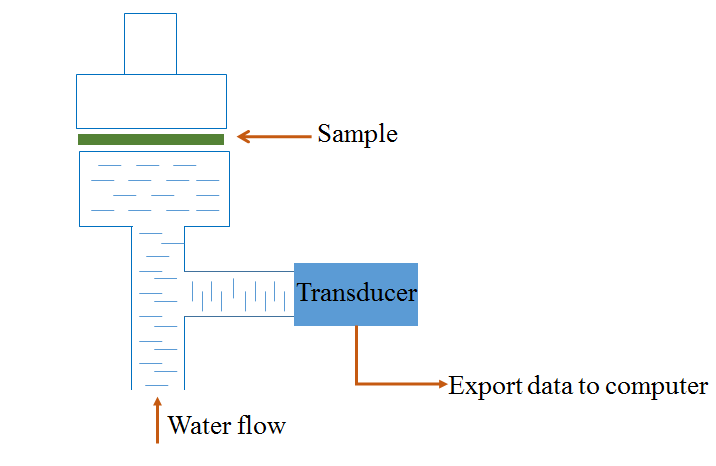


Figure S11. Illustration of breakthrough pressure test.

The pressure ~ time curve was recorded by a pressure sensor lined with computer. The max P that liquid penetrates through the membrane was recorded as breakthrough pressure.


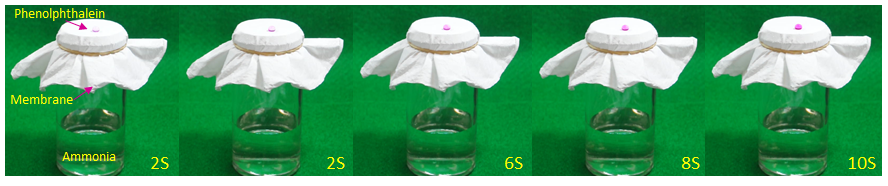


Figure S12. Illustration of ammonia vapor passing through the FAS-modified SBS fibrous membrane and causes colorization reaction of a phenolphthalein-containing water drop.

To illustrate the good permeability of the fibrous membrane, we placed an ammonia solution to a vial and then sealed it with a piece of FAS modified SBS fibrous membrane. Upon dropping phenolphthalein-containing water on the membrane, the droplet stayed stably on the membrane surface. In 3 seconds, the droplet turned red.
